# Matriptase regulates c-Met mediated proliferation and invasion in inflammatory breast cancer

**DOI:** 10.18632/oncotarget.11262

**Published:** 2016-08-12

**Authors:** Gina L. Zoratti, Lauren M. Tanabe, Thomas E. Hyland, Michael J. Duhaime, Éloïc Colombo, Richard Leduc, Eric Marsault, Michael D. Johnson, Chen-Yong Lin, Julie Boerner, Julie E. Lang, Karin List

**Affiliations:** ^1^ Department of Oncology, Wayne State University School of Medicine and Barbara Ann Karmanos Cancer Institute, Detroit, MI 48201, USA; ^2^ Department of Pharmacology, Wayne State University School of Medicine and Barbara Ann Karmanos Cancer Institute, Detroit, MI 48201, USA; ^3^ Department of Pharmacology, Faculty of Medicine and Health Sciences, Université de Sherbrooke, Sherbrooke, QC J1H5N4 Canada; ^4^ Lombardi Comprehensive Cancer Center, Department of Oncology, Georgetown University, Washington DC 20057, USA; ^5^ Department of Surgery, Norris Comprehensive Cancer Center, University of Southern California, Los Angeles, CA 90033, USA

**Keywords:** inflammatory breast cancer, matriptase, type II transmembrane serine proteases

## Abstract

The poor prognosis for patients with inflammatory breast cancer (IBC) compared to patients with other types of breast cancers emphasizes the need to better understand the molecular underpinnings of this disease with the goal of developing effective targeted therapeutics. Dysregulation of matriptase expression, an epithelial-specific member of the type II transmembrane serine protease family, has been demonstrated in many different cancer types. To date, no studies have assessed the expression and potential pro-oncogenic role of matriptase in IBC. We examined the functional relationship between matriptase and the HGF/c-MET signaling pathway in the IBC cell lines SUM149 and SUM190, and in IBC patient samples. Matriptase and c-Met proteins are localized on the surface membrane of IBC cells and their expression is strongly correlated in infiltrating cancer cells and in the cancer cells of lymphatic emboli in patient samples. Abrogation of matriptase expression by silencing with RNAi or inhibition of matriptase proteolytic activity with a synthetic inhibitor impairs the conversion of inactive pro-HGF to active HGF and subsequent c-Met-mediated signaling, leading to efficient impairment of proliferation and invasion of IBC cells. These data show the potential of matriptase inhibitors as a novel targeted therapy for IBC, and lay the groundwork for the development and testing of such drugs.

## INTRODUCTION

Inflammatory breast cancer (IBC) is a rare and aggressive form of invasive breast cancer accounting for 2–4%, or about 4,000 breast cancer cases annually in the United States. IBC is characterized by rapid progression, local and distant metastases, younger age of onset, and lower overall survival compared with other breast cancers, with a 5-year survival rate of 25–40% when treated with neoadjuvant chemotherapy, mastectomy, and postmastectomy radiation. IBC patients often present with a breast that looks inflamed due to extensive lymphovascular invasion of tumor emboli which block lymphatic drainage from the breast, but no palpable tumor. The rapid development of metastases in IBC results from high proliferative rates and potent ability for angiogenesis and lymphangiogenesis. Furthermore, 20–40% of IBC cases are triple-negative breast cancers (TNBC) which excludes hormone therapy and HER2 targeting as treatment options [1–3].

Matriptase is an epithelial-specific member of the type II transmembrane serine protease family and dysregulation of matriptase has been demonstrated in many different cancer types [4–7]. Extracellular proteases have long been associated with cancer progression because of their ability to degrade extracellular matrices, which promotes invasion and metastasis. This view has been expanded with the findings that proteolysis regulates multiple steps of tumor progression including proliferation, differentiation, apoptosis, and invasion. One concept in protease mechanistic research is that proteolytic modifications of targets, including activation of growth factors, are critically involved in carcinogenesis through activation of oncogenic signaling pathways.

The pro-form of the hepatocyte growth factor (pro-HGF) requires specific proteolytic cleavage to be biologically active. HGF is the ligand of the proto-oncogene receptor tyrosine kinase, c-Met, and when matriptase-cleaved HGF is bound to its receptor, the activated signaling pathway elicits downstream pro-tumorigenic events including cell proliferation, migration, morphogenesis, and invasion [8–12]. To date, no published studies have assessed the expression and potential pro-cancerous role of the matriptase/HGF/c-Met signaling axis in IBC. Here, we present evidence that matriptase may be considered a novel therapeutic target for IBC.

## RESULTS

### Correlation between matriptase and c-Met expression in IBC patient samples

**Figure 1:**
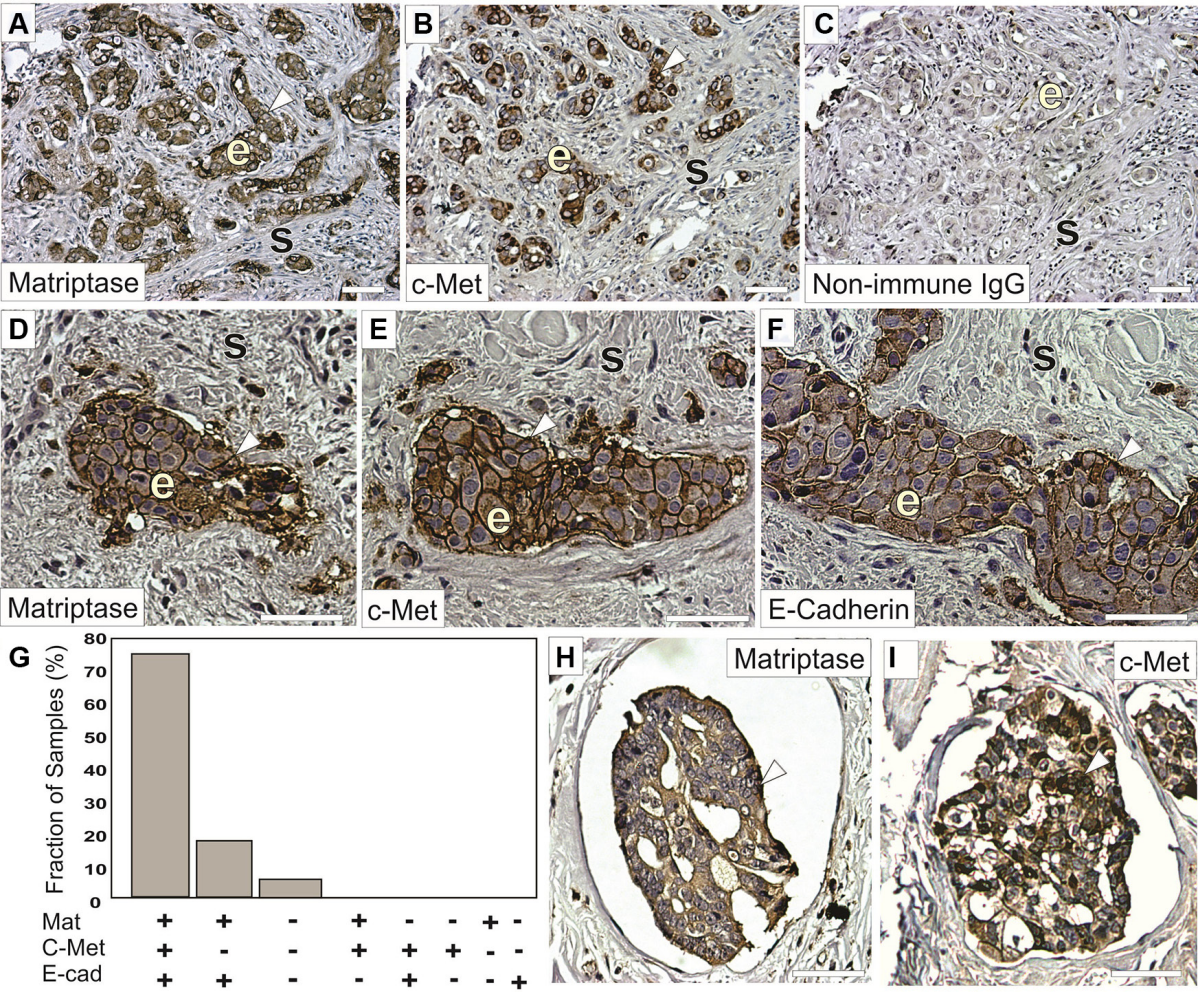
Matriptase and c-Met protein are co-expressed in IBC Representative staining of serial sections of IBC using a rabbit anti-matriptase antibody (**A**, **D**, **H**) a rabbit-c-Met antibody (**B**, **E**, **I**) or a mouse anti-E-cadherin antibody (**F**). Primary antibodies were substituted with non-immune IgG as negative controls (**C**). Matriptase, c-Met, and E-cadherin proteins are primarily localized on cell surfaces (brown staining, arrow heads) of invasive epithelia derived IBC cells (indicated with ‘e’) with no significant staining in the mesenchymal/stromal compartments (indicated with ‘s’) and display highly similar expression patterns. (**G**) Expression of matriptase, c-Met, and E-cadherin in 22 human IBC cases. Bars depict the frequency of samples expressing matriptase, c-Met, and E-cadherin (17/22), matriptase and e-cadherin (4/22), neither protein (1/22), no sample displayed the other expression combinations indicated. (**H**–**I**) Representative staining for matriptase (H) and c-Met (I) (brown staining, arrow heads) in dermal lymphatic emboli. Both matriptase and c-Met are primarily localized on cell surfaces and in the cytoplasm of IBC cells. Tissues were counterstained with haematoxylin (blue/grey). Scale bars, 50 μm.

Matriptase and c-Met are cell surface proteins that are primarily expressed in epithelial cells. We hypothesized that a prerequisite for matriptase and c-Met to be functionally linked *in vivo* is that they are expressed in close proximity to each other, on either the same cell or neighboring cells. Therefore, an important component to elucidate the roles of matriptase and c-Met in IBC is to determine their expression and localization in IBC patient samples and IBC cultured cells. To ensure specific staining in immunohistochemistry (IHC), we used antibodies that we had previously validated [7]. Another transmembrane protein expressed on the surface of cancer cells, E-cadherin, is believed to function as a tumor suppressor in some invasive breast carcinomas and its expression is frequently lost during cancer progression. Interestingly, it has been observed that E-cadherin is retained in the majority of IBC cases [19–21]. To assess whether a similar E-cadherin expression profile is observed in our sample collection, we performed parallel E-cadherin IHC. Of the 22 IBC patient samples analyzed, the majority (17/22, 77%) displayed expression of matriptase, c-Met, and E-cadherin in infiltrating cancer cells and in the cancer cells of peritumoral and dermal lymphatic emboli (Figure 1 and Supplementary Figure S1). Information about patient samples can be found in “Materials and Methods” and in Table 1. Furthermore, the staining patterns for all three proteins were strikingly similar, consistent with co-localization of the protease, the growth factor receptor, and the adherens junction protein in IBC (Figure 1). No significant staining was observed in tumor stromal compartments. Samples from four patients (18%) expressed both matriptase and E-cadherin but not c-Met, whereas one sample expressed no detectable matriptase, c-Met or E-cadherin. Importantly, the vast majority (21/22, 95%) of the samples retained E-cadherin expression. All E-cadherin expressing samples expressed matriptase and vice versa.

**Table 1:** Expression of matriptase, c-Met, and E-cadherin in IBC patient samples

Sample ID	Surg/BX	Treatment	Matriptase	c-Met	E-cadherin
1	S	pre-op Chemotherapy	+	+	+
2	S	pre-op Chemotherapy	+	+	+
3	S	pre-op Chemotherapy	+	+	+
4	S	pre-op Chemotherapy	+	+	+
5	S	pre-op Chemotherapy	+	+	+
6	S	pre-op Chemotherapy	+	+	+
7	S	pre-op Chemotherapy	+	+	+
8	S	pre-op Chemotherapy	+	+	+
9	S	No info	−	−	−
10	S	pre-op Chemotherapy	+	−	+
11	S	pre-op Chemotherapy	+	+	+
12	S	pre-op Chemotherapy	+	+	+
13	BX	No treatment	+	+	+
14	S	No info	+	+	+
15	S	pre-op Chemotherapy	+	−	+
16	S	pre-op Chemotherapy	+	−	+
17	S	pre-op Chemotherapy	+	+	+
18	BX	No treatment	+	+	+
19	S	pre-op Chemotherapy	+	+	+
20	S	pre-op Chemotherapy	+	+	+
21	S	pre-op Chemotherapy	+	+	+
22	BX	No treatment	+	−	+

Protein expression of matriptase, c-Met, and E-cadherin determined by IHC. “+” indicates clear expression in the majority of cancer cells and “−“indicates no detectable staining above background (compared to serial sections incubated with a non-immune antibody). “S” indicates that the sample was acquired during surgery and “BX” indicates biopsy acquired sample.

### Subcellular localization to the cell surface membrane in IBC cells

**Figure 2:**
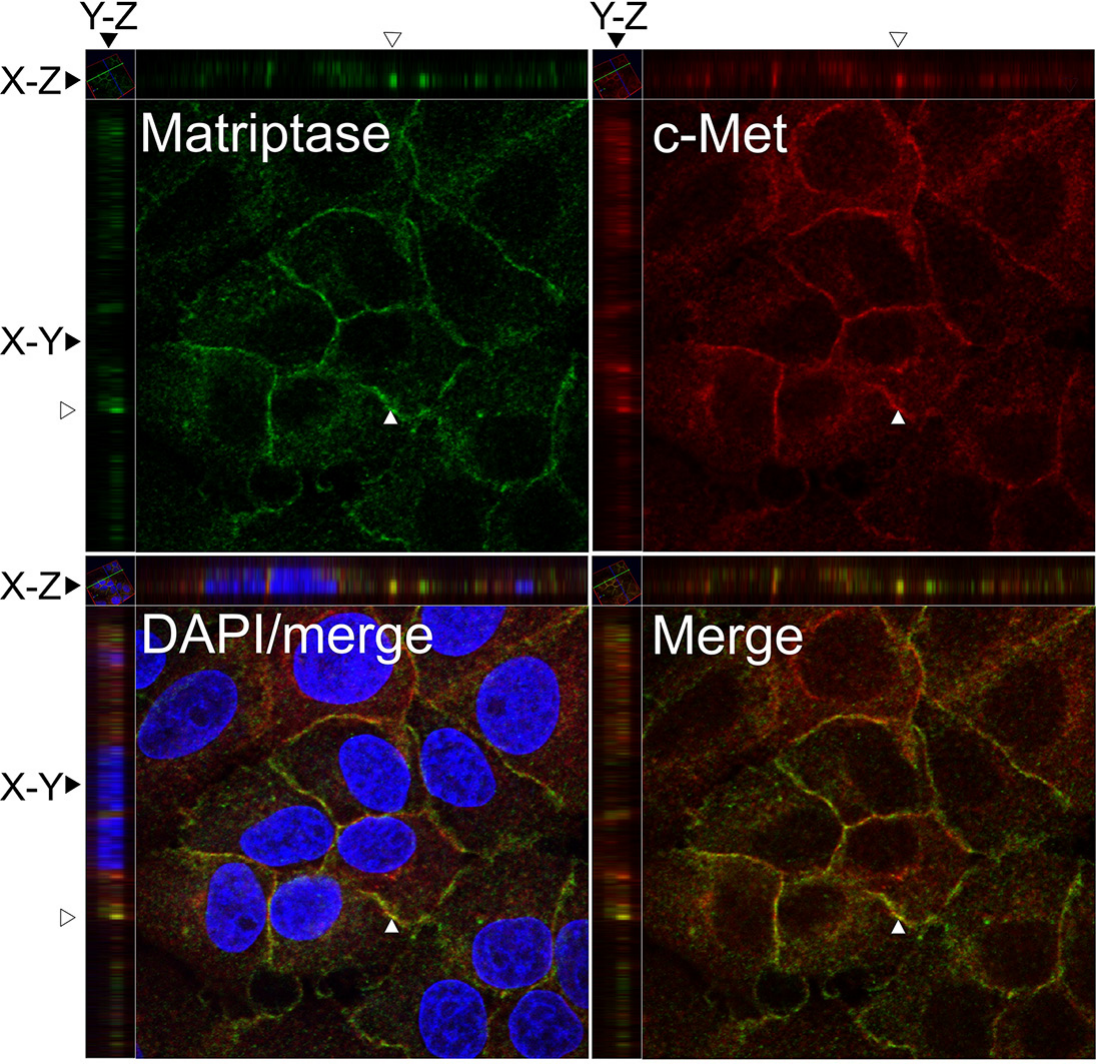
Matriptase and c-Met proteins are co-expressed in IBC cell lines SUM149 cells were permeabilized and incubated with mouse anti-matriptase (M24) and rabbit anti-C-Met, followed by fluorophore-conjugated secondary antibodies. The cells were visualized by confocal fluorescence microscopy: Alexa Fluor 488, matriptase (upper left panel); Alexa Fluor 546, c-Met (upper right panel); DAPI/matriptase/c-Met merged (lower left panel) and matriptase/c-Met merged (lower right panel). Images were analyzed with Volocity^™^ software. A representative orthogonal section from the original Z-stack was used to assess co-localization (yellow) in XY, XZ, and YZ planes. The Y and Z plane views were taken at the position indicated with an open arrow head. Scale bars, 10 μm.

To further study the localization of matriptase and c-Met at the subcellular level, the endogenous proteins in the IBC line SUM149 were visualized by immunocytochemistry and confocal imaging (Figure 2). Matriptase and c-Met were mainly expressed on the cell surface, as expected, based on the membrane topology of both proteins. Analysis of confocal images shows extensive localization of both matriptase and c-Met on the cell surface membrane with weaker cytoplasmic staining.

Taken together with the results above, matriptase and c-Met are co-expressed in the majority of IBC patient samples analyzed and localize on the surface of IBC cells. Furthermore, the majority of IBC patient samples express E-cadherin.

### Matriptase silencing abrogates pro-HGF mediated c-Met signaling in human IBC cell lines

To examine the molecular and functional relationship between matriptase and c-Met in IBC, we employed cell culture models in both 2D and 3D. SUM149 (ER-/PR-/HER2-) and SUM190 (ER-/PR-/HER2+) cells were originally isolated from primary inflammatory invasive ductal carcinoma and display characteristics commonly found in IBC tumors, including expression of E-cadherin [20–23].

**Figure 3:**
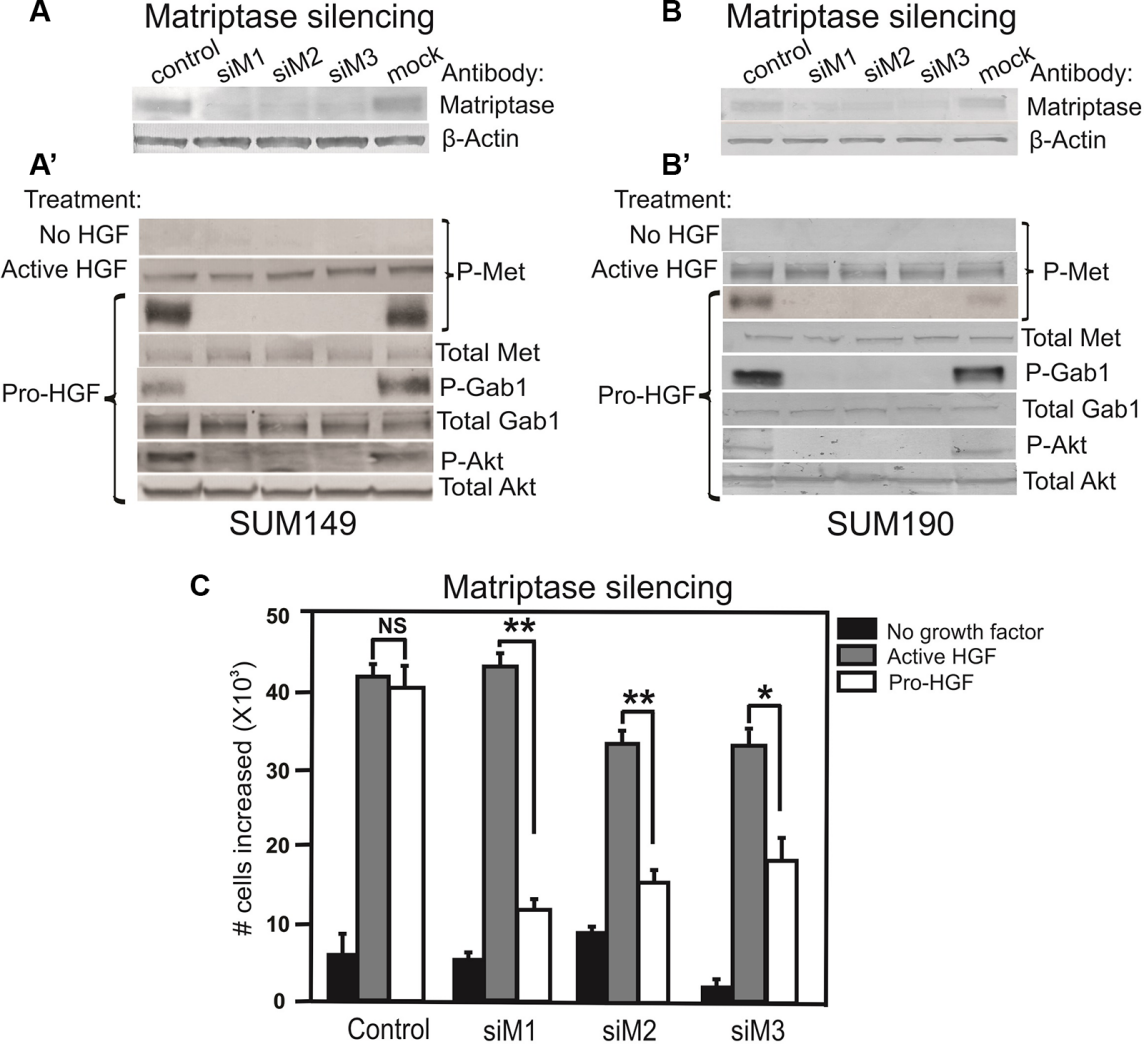
Matriptase is essential for activation of the pro-HGF/c-Met signaling pathway and proliferation in IBC cells RNAi silencing of matriptase in SUM149 cells (**A**) or SUM190 cells (**B**) with three independent non-overlapping synthetic RNA duplexes (siM1, siM2, and siM3). A %GC matched RNA duplex was used as negative control as well as a mock where no RNA duplex was added. (A', B') Cells were serum starved and exposed to vehicle, active HGF (2 min), or pro-HGF (15 min) as indicated. Cell lysates were separated by SDS-PAGE and analyzed for the presence of activated c-Met and its downstream targets by western blotting. As expected, no phosphorylation of c-Met was observed when no HGF was added, whereas addition of pre-cleaved, active HGF resulted in phosphorylation of c-Met in all samples. When pro-HGF was added, no phosphorylation of c-Met or its downstream targets, Gab1 and AKT, was observed in cells where matriptase was silenced with any of the three RNA duplexes. (**C**) SUM149 cells were grown on culture plates and matriptase was silenced using the three synthetic RNA duplexes mentioned above. The cells were serum starved and left untreated (black bars) or exposed to either active HGF (gray bars) or pro-HGF (white bars) for 24 h and counted. Bar graphs show the proliferation at 24 h after stimulation (*P < 0.05, **P <0.01). Error bars represent S.D. of triplicates within one assay. Data are representative of four independent experiments.

Analysis of cellular proteins from SUM149 and SUM190 by western blotting showed that matriptase and c-Met are expressed in both cell lines (Figure 3A, 3A', 3B, 3B' and Supplementary Figure S2) and that stimulation with pro-HGF/HGF is required for c-Met activation since no phosphorylated c-Met was detected in the absence of pro-HGF/HGF (Figure 3A' and 3B', top panels “No HGF”). Based on these characteristics, the two IBC cell lines were deemed appropriate for further studies on matriptase/c-Met function.

It has been demonstrated that matriptase can cleave and activate the pro-form of HGF [24]. However, no studies describing a role for matriptase as a pro-HGF activating protease in IBC have been reported. To determine whether matriptase regulates c-Met signaling via activation of pro-HGF, SUM149 and SUM190 cells were stimulated with pro-HGF or active HGF and the phosphorylation/activation state of c-Met and its downstream signaling proteins, Gab1 and AKT, were determined by western blot analysis. HGF is secreted in its inactive pro-form by stromal cells in both human and mice, including stromal mammary fibroblasts and macrophages [25–27]. The source of pro-HGF in this experimental set-up was human mammary fibroblasts expressing pro-HGF [16]. To directly assess the role of matriptase for pro-HGF activation, experiments using RNAi mediated silencing of matriptase were performed. Silencing of matriptase with three independent non-overlapping synthetic RNA duplexes was achieved in SUM149 and SUM190 cells (Figure 3A and 3B). Addition of pro-HGF to matriptase sufficient control cells resulted in robust activation of c-Met and the downstream targets Gab1 and AKT (Figure 3A' and 3B', “pro-HGF”). In contrast, matriptase silencing led to greatly reduced or undetectable levels of c-Met pathway activation in both SUM149 and SUM190 cells. Importantly, the matriptase silenced cells displayed unaltered activation of c-Met upon stimulation with pre-cleaved active HGF, demonstrating that the impaired response to pro-HGF is caused by the lack of matriptase-mediated cleavage, while the cells remain fully HGF/c-Met signaling competent (Figure 3A' and 3B', second panel, “active HGF”).

To assess the functional consequences of abrogating the matriptase-meditated c-Met signaling in IBC cells, a proliferation assay was performed. As described above, matriptase was silenced in SUM149 with three different synthetic RNA duplexes and cells were stimulated with pro-HGF or active HGF. As expected, there was no significant proliferation difference in matriptase sufficient control cells treated with active HGF or pro-HGF. In contrast, proliferation in matriptase silenced cells was significantly impaired in response to pro-HGF (Figure 3C, white bars) in comparison to the response observed with addition of pre-cleaved active HGF (Figure 3C, grey bars).

### Matriptase silencing impairs pro-HGF/c-Met mediated invasion in human IBC cells

**Figure 4:**
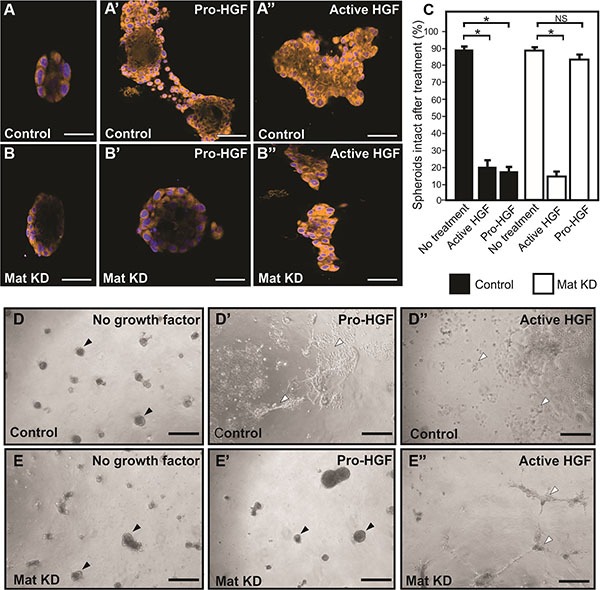
Matriptase mediates pro-HGF induced invasion of IBC in 3D culture Matriptase was silenced in SUM149 cells (Mat KD) using synthetic RNA duplexes (B, B', B″). A %GC matched duplex was used as negative control (A, A', A″). Cells formed spheroids in 3D rBM overlay culture (**A**, **B**) before addition of either pro-HGF (A', B') or active HGF (A″, B″) and imaged by confocal microscopy. Cells were labeled with Cell Tracker Orange and nuclei with Hoechst (blue). When no growth factors were added, control and Mat KD spheroids remained intact. In contrast, addition of pro-HGF induced extensive invasive outgrowths in matriptase sufficient control cells (A') whereas the majority of Mat KD spheroids remained intact (B'). Both control and Mat KD spheroids responded to active two-chain HGF (A″, B″). Scale bars, 50 μm (**C**) 3D rBM cell cultures were scored for the number of intact control spheroids (black bars) and intact Mat KD spheroids (white bars) when left untreated or exposed to either active HGF or pro-HGF. Data represent mean of the number of intact spheroids before treatment relative to the number after treatment (*P < 0.05). Error bars represent S.D. of triplicates within one assay. Data are representative of four independent experiments. (**D**–**E**) Low magnification photos of independent experiment performed as above. Pro-HGF (D', E'), HGF (D″, E″), or no growth factor (D, E). When no HGF was added, control and Mat KD spheroids remained intact (D, E) (black arrowheads). In contrast, addition of pro-HGF induced extensive scattering and invasion in matriptase sufficient control cells (D') (indicated with open arrowheads) whereas the majority of spheroids in Mat KD cells remained intact (E'). Both control and Mat KD responded to active two-chain HGF (D″, E″) Scale bars, 200 μm.

The standard 2D cell culture models described above are optimal for biochemical analysis of matriptase-mediated c-Met signaling in IBC. However, additional models of IBC are beneficial to study the functional importance of this pathway. Breast cancer cells grown in 3D cultures are believed to better recapitulate *in vivo* morphology and invasion than cells grown on tissue culture plates. SUM149 cells were transiently silenced using matriptase RNA duplexes or control duplexes and transferred to 3D culture containing growth factor depleted reconstituted basement membrane (rBM) (Figure 4). All cells formed 3D spheroid structures within 24 hours in this model (Figure 4A, 4B, 4D, 4E no HGF). Upon stimulation with pro-HGF or active HGF for 24 hours, matriptase sufficient control cells displayed pronounced invasive growth characterized by branching cellular structures and single cells with a flattened spindle-like appearance (Figure 4A', 4D'). In contrast, matriptase silencing (Mat KD) rendered the cells unresponsive to pro-HGF (Figure 4B', 4E'), thus, keeping the vast majority of the cells confined in compact spheroid structures (Figure 4C). Addition of pre-cleaved active HGF restored the ability of matriptase-deficient cells to display a similar phenotype to control cells, suggesting that the c-Met signaling pathway remained intact (Figure 4B″, 4E″). These data demonstrate that matriptase potentiates the invasive capacity of IBC cells by activation of pro-HGF.

### Inhibition of matriptase proteolytic activity impairs IBC cell proliferation upon pro-HGF stimulation

**Figure 5:**
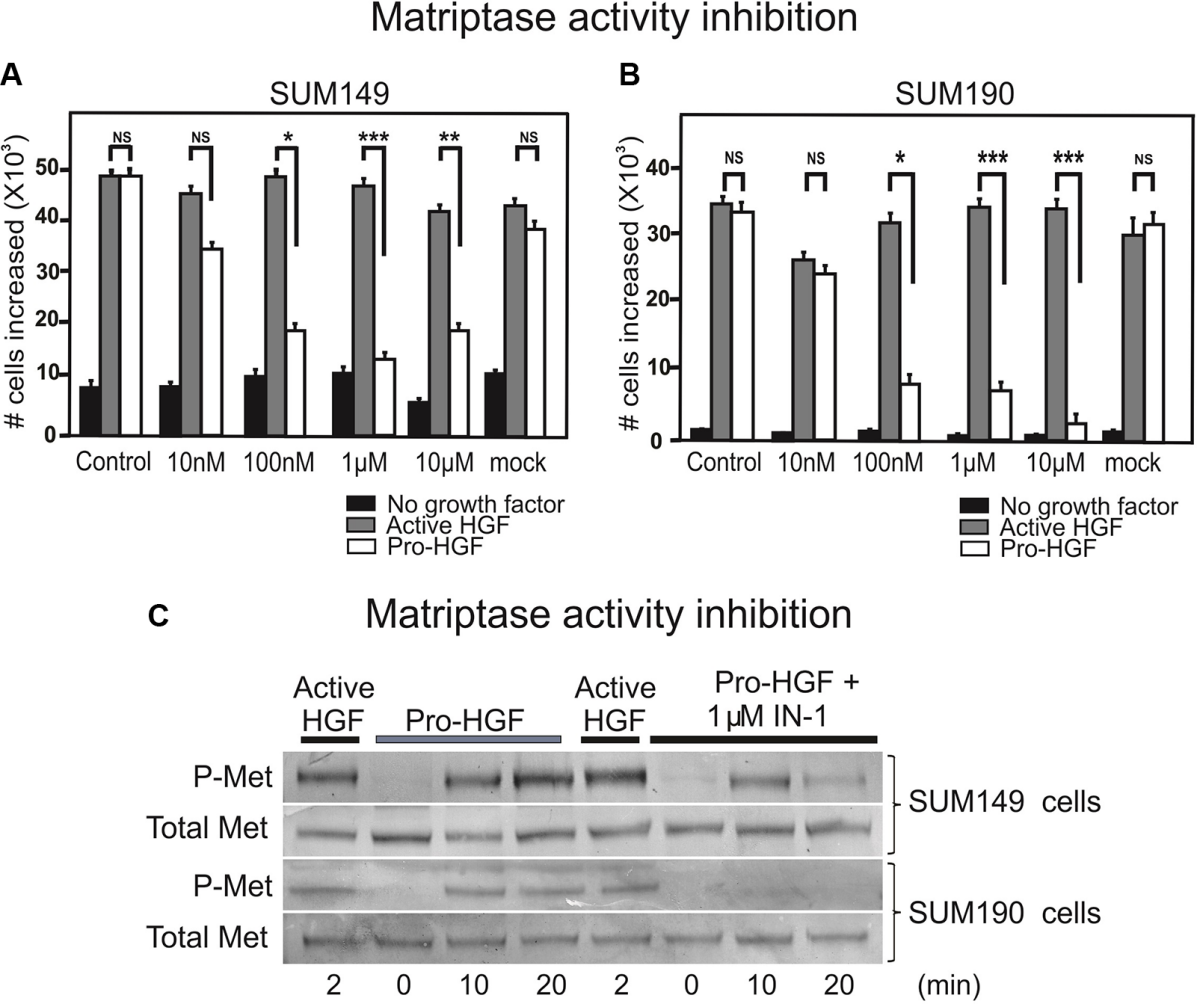
Inhibition of matriptase proteolytic activity impairs IBC cell proliferation upon pro-HGF stimulation SUM149 (**A**) and SUM190 (**B**) cells were serum starved and IN-1 was added at 10 nM, 100 nM, 1 μM, or 10 μM concomitantly with active HGF (gray bars), pro-HGF (white bars), or no growth factors (black bars) for 24 h and cells were counted. In the mock control vehicle (PBS) was added. Graphs show the increase in cell numbers at 24 h after stimulation (*P < 0.05, **P < 0.01, ***P < 0.001). Error bars represent S.D. of triplicates within one assay. Data are representative of three independent experiments. (**C**) SUM149 cells (upper panels) and SUM190 cells (lower panels) cells were serum starved and pre-incubated with 1 μM IN-1 synthetic matriptase inhibitor for 30 min before exposure to either active HGF or pro-HGF. Cells were lysed at the indicated time points after growth factor exposure and the levels of total c-Met and phospho-c-Met were determined by western blotting.

Based on our findings that matriptase is critically involved in IBC cell proliferation and invasion, this protease represents a potential target for drug development. To determine whether abolishing matriptase proteolytic activity would be applicable, we tested a matriptase inhibitor, IN-1, in the proliferation assay using the SUM 149 and SUM190 lines. IN-1 is highly selective for matriptase versus other related proteases inclucding hepsin (100-fold), matriptase-2 (300-fold), TMPRSS11D (764-fold), thrombin (> 30000-fold), and furin (no inhibition) [15]. IN-1 efficiently inhibited the proliferative response to pro-HGF in a dose dependent manner in both SUM149 cells (Figure 5A, white bars) and SUM190 cells (Figure 5B, white bars) with significant inhibition observed at 100 nM. No inhibitory effect was observed in response to pre-activated HGF, indicating that the matriptase-mediated pro-HGF conversion is inhibited by IN-1 (Figure 5A, 5B, gray bars). Growth inhibition by IN-1 in both cell lines was due to abrogation of c-Met activation as demonstrated by western blotting (Figure 5C). Matriptase inhibition led to greatly reduced (SUM149, 5C upper panels) or undetectable (SUM190, 5C lower panels) levels of activated c-Met, whereas the response to pre-cleaved active HGF was unaltered. Together, these results demonstrate that silencing of matriptase expression, or inhibition of matriptase catalytic activity, leads to decreased pro-HGF activation and subsequent c-Met signaling pathway activation, ultimately causing impairment of the proliferative and invasive response in IBC cells. Also, the results suggest that matriptase is a druggable protease that may represent a novel target for pharmaceutical intervention in IBC.

## DISCUSSION

Because of the rarity of IBC and the inherent difficulty in obtaining tumor tissue from patients, few studies have been performed to map the molecular characteristics of this disease. Understanding the distinct pathology of IBC and the molecular mechanisms involved in the rapid disease progression will likely provide insights to aid the discovery of new treatments. The present study includes novel expression, mechanistic, and functional studies of the matriptase/HGF/c-Met epithelial-mesenchymal protease-mediated signaling axis that may represent a future target for therapeutic intervention in IBC.

An important component to elucidate the roles of matriptase and c-Met in IBC was to determine their expression and localization in IBC patient samples. Immunohistochemistry studies of serial sections demonstrated expression of both matriptase and c-Met in infiltrating cancer cells and in the cancer cells of lymphatic emboli in the majority of IBC patients. The high frequency of c-Met positive samples is in accordance with a previous study [28]. Furthermore, matriptase and c-Met are co-localized on the cell surface in cultured IBC cells.

It has been demonstrated that matriptase can cleave and activate the pro-form of HGF which is a pleiotropic, paracrine growth factor and key mediator of cell migration, proliferation, survival, motility, and morphogenesis in epithelial cells [29]. Cleaved, activated HGF exerts its effects by binding to the proto-oncogene tyrosine kinase receptor, c-Met, that elicits pro-tumorigenic downstream signaling events [29]. Since a key post-translational regulation mechanism of HGF/c-Met signaling is the proteolytic activation of pro-HGF, the identification of the physiological activator as a potential target for therapeutic intervention in IBC is critical. We demonstrate that matriptase regulates c-Met signaling via activation of pro-HGF in SUM149 and SUM190 IBC cells. Functionally, silencing of matriptase expression in IBC cells causes severe impairment of proliferation and invasion. These findings prompted us to test whether a synthetic matriptase inhibitor could inhibit matriptase-mediated c-Met activation and proliferation. Indeed, the IN-1 inhibitor efficiently inhibits proliferation in both IBC cell lines with significant inhibition observed at 100 nM. It should be mentioned, that two other members of the type II transmembrane serine protease family, hepsin and TMPRSS13, which have previously been reported as being able to cleave and activate pro-HGF *in vitro* [30–32], were readily detectable by western blot analysis in a variety of breast cancer cells lines (unpublished data), which included the SUM149 and SUM190 lines used in this study. Furthermore, HGF activator (HGFA), a soluble protease, has been reported to be expressed in multiple breast cancer cell lines and in human breast tumors [33]. Since no or low residual pro-HGF-activating activity was detected upon matriptase silencing, it is unlikely that these proteases act as efficient pro-HGF activators on the cell surface of IBC cells in the cell culture models used in the present study. It cannot be ruled out however, that pro-HGF is activated by several proteases in IBC *in vivo*.

The IHC analysis revealed a high frequency of samples expressing E-cadherin, which is in agreement with previously published studies [20–21]. We have previously shown a correlation between matriptase and E-cadherin expression in breast cancer cells of diverse origin representing a variety of molecular subtypes [14]. Thus, matriptase and E-cadherin are expressed in IBC cells and in non-IBC cells that have preserved epithelial characteristics. Interestingly, neither matriptase nor E-cadherin is detectable in cell lines that have gained a mesenchymal-like phenotype, with high motility and loss of cell–cell adhesion [14]. The loss of E-cadherin, with a switch to N-cadherin expression and other mesenchymal markers, is considered a hallmark of epithelial-to-mesenchymal transition (EMT). Since loss of E-cadherin has been shown to promote invasion and metastasis in many different carcinomas, it has long been considered a paradox that IBC cells, despite their aggressiveness, maintain E-cadherin expression in the primary tumor and tumor emboli [21]. In fact, it has been shown that E-cadherin is overexpressed in IBC and that this contributes to the formation of the lymphovascular embolus, a cellular structure efficient at metastasis [19]. Recent studies have shown that reduced expression of E-cadherin leads to a dramatic reduction of the *in vivo* growth capability of IBC tumor cells [21]. The authors propose that E-cadherin plays a role in cellular plasticity during the reversible processes of EMT and mesenchymal-to-epithelial transition (MET). Cancer cells retain tight control of EMT features such as motility and invasion, while maintaining the capacity to display epithelial features such as E-cadherin expression to favor tumor growth and the establishment of metastatic tumors once the cells have reached their site [21]. Our data suggest that matriptase, via activation of the c-Met pathway, may be a player in this complex regulation of cellular plasticity by stimulating both proliferation and invasion of IBC cells. Whether matriptase plays additional non-HGF/c-Met mediated roles in IBC tumorigenicity and whether the observed link between expression/loss of matriptase and E-cadherin in IBC is correlational or causal in breast cancer remains to be explored.

Several drugs that target c-Met, including antibodies that bind to c-Met and compete with pro-HGF/HGF binding, and c-Met kinase inhibitors have been developed. Currently, there are a number of ongoing phase 2 clinical trials using various c-Met drugs in patients with TNBC, including IBC. Some of the c-Met kinase inhibitors are highly selective for c-Met, whereas others target additional receptors such as the c-Met-VEGFR-2 inhibitor, XL184 (cabozantinib), and the cMet-ALK (anaplastic lymphoma kinase) inhibitors, X-396 and X-376 (crizotinib). In preclinical 3D cell culture models and mouse models of TNBC growth, invasion, and metastasis, it was demonstrated that cabozantinib significantly inhibits TNBC progression and metastasis [34]. It was observed that c-Met positive TNBC cells were significantly inhibited with cabozantinib, whereas c-Met negative cells were not, suggesting that c-Met signaling is the critical pathway in this TNBC cell model that is targeted by XL184 treatment [34]. Another study assessed the response of IBC cell lines to treatment with crizotinib that inhibits c-Met and ALK [31]. The majority of IBC patient samples analyzed (20/25, 80%) had either increased ALK copy number, low level ALK gene amplification, or ALK gene expression. Crizotinib was cytotoxic to all ALK expressing cell lines tested. In addition, SUM149 cells were also sensitive to crizotinib. Since this cell line displays very low levels of ALK expression, the authors suggest that the drug effect is due to protein kinase inhibition of c-Met [35]. Targeting multiple pathways and combining inhibitors with different modes of action, including proteases that activate tumorigenic growth factors, may circumvent resistance to kinase inhibitors [29].

In conclusion, we have demonstrated that targeting the pro-HGF activating protease, matriptase, in IBC efficiently inhibits pro-oncogenic c-Met signaling, and that future studies testing potent and highly selective matriptase inhibitors and their potential as novel targeted drugs in IBC as well as TNBC may be a promising new therapeutic strategy.

## MATERIALS AND METHODS

### Human IBC tissue

De-identified cases of normal breast tissue and inflammatory infiltrating ductal carcinoma were identified by a breast surgeon (J.E.L) via the University of Arizona Cancer Center Tissue Acquisition and Cellular/Molecular Analysis Core. A board certified pathologist verified the assignment of IBC versus non-IBC status for all cases by inspection of surgical pathology slides and reports. The IBC specimens were acquired from a punch biopsy before treatment or after neoadjuvant chemotherapy at the time of modified radical mastectomy (Table 1). Of the IBC patients, 36.4% (*n* = 8/22) were confirmed to have metastatic breast cancer as subsequent distant recurrences. Of patients with distant metastatic disease, 87.5% (*n* = 7/8) died of breast cancer. 45.4% (*n* = 10) were lost to follow-up and 1 had metastatic non-small cell lung cancer. Only 13.6% (*n* = 3) patients were confirmed not to have metastatic disease after 5 years of follow-up. The small sample size precludes a statistical analysis of matriptase/c-met expression in regards to survival, particularly when considering that survival outcomes in IBC patients typically is far inferior to non-IBC patients. The high rates of loss to follow-up are consistent with the fact that University Medical Center is a tertiary care referral institution serving a predominantly rural, underserved patient population. Patients with IBC, a rare disease, were likely to be treated initially at our institution but later preferred to complete routine follow-up care in their own communities. Representative H&E stained and 5 unstained slides cut as 3-micron serial sections were provided via an IRB approved tissue biorepository (#06-0609-04) and transferred to Wayne State University for immunohistochemical analysis under an approved institutional IRB Exemption (# 2011–156).

### Immunohistochemistry

Tissue slides were deparaffinized with xylene and hydrated with graded ethanol solutions. Antigen retrieval was performed using citrate buffer, pH 6.0 (Bethyl Laboratories, Montgomery, TX) and incubation for 1 h in a 73^°^C water bath. The slides were blocked with 2% bovine serum albumin in PBS, and immunostained overnight at 4^°^C. Primary antibodies were rabbit anti-matriptase (Calbiochem/EMD Millipore, San Diego, CA), rabbit anti-c-Met (Leica Microsystems Inc., Buffalo Grove, IL), and mouse anti-E-cadherin (Pharmingen/BD Biosciences, San Jose, CA). As negative controls, non-immune mouse IgG (Sigma, St. Louis, MO) or non-immune rabbit IgG (NeoMarkers, Fremont, CA) were used. Bound antibodies were visualized using biotin-conjugated anti-rabbit or anti-mouse (Vector Laboratories, Burlingame, CA) secondary antibodies and a Vectastain ABC kit (Vector Laboratories). 3,3′-diaminobenzidine (DAB) was used as substrate (Sigma, St. Louis, MO) and arrays were counterstained with hematoxylin. All microscopic images were acquired on a Zeiss Scope A.1 using digital imaging.

### Human breast cancer cell lines

The SUM149 and SUM190 cell lines were a gift from Dr. Stephen Ethier (Medical University of South Carolina, Charleston, SC). SUM149 cells were grown in 5% IH media (Ham's F-12 media, supplemented with 5% FBS, 1μg/ml hydrocortisone, and 5 μg/ml insulin). SUM190 cells were grown in SFIH media (Ham's F-12 media, supplemented with 1 μg/ml hydrocortisone, 5 μg/ml insulin, 5 mM ethanolamine, 10 mM HEPES, 5 μg/ml transferrin, 10 nM triodo-thyronine, 50 μM sodium selenite, and 5% BSA).

### Immunocytochemistry

SUM149 cells were fixed with 10% neutral-buffered zinc formalin (Z-fix) (Anatech, Battle Creek, MI) for 20 min, blocked in 5% BSA with 0.1% Triton X-100 for 1 hr, and incubated with rabbit anti-c-Met (Cell Signaling Technology, Beverly, MA) and mouse monoclonal anti-matriptase M24 antibody [13] overnight at 4^°^C. The following day, cells were washed with PBS, and incubated with anti-rabbit 546 or anti-mouse 488 antibodies (Invitrogen, Carlsbad, CA) in blocking solution for 2 hrs at RT. Cells were washed with PBS and mounted with Prolong gold with DAPI (Invitrogen). Confocal images were acquired on the Zeiss LSM 780 scope at the Microscopy Imaging and Cytometry Resources Core at Wayne State University School of Medicine. Images were analyzed using Volocity^™^ software.

### RNAi silencing

For matriptase knockdown in SUM149 and SUM190 IBC cells, three independent Stealth RNAi^™^ siRNA duplexes targeting matriptase (siM1-ST14HSS186125, siM2-ST14HSS186126, siM3- ST14HSS110268), as well as %GC matched negative controls were used as previously described (Invitrogen) [14]. Two days after transfection, the cells were serum starved and used in the pro-HGF/HGF activation assay, in the 3D invasion assay, and in the proliferation assay described below.

### Matriptase synthetic inhibitor

A selective, slow, tight-binding inhibitor of matriptase containing a ketobenzothiazole serine trap (IN-1) was designed based on the auto-catalytic domain (RQAR) of matriptase. Details on the synthesis and characteristics of the inhibitor have been described previously [15]. IN-1 was solubilized in PBS and used at concentrations ranging from 10 nM to 10 μM.

### Pro-HGF/SF activation assays

Pro-HGF/SF was produced in the immortalized human fibroblasts cell line, RMF-HGF, as described [16, 17]. In order to estimate the concentration of each preparation and ensure quality, western blot analysis was performed using a goat anti-HGF primary antibody (R&D Systems, Minneapolis, MN) with recombinant human recombinant active HGF (R&D Systems) as a standard. Human IBC cell lines were plated in 6-well plates, grown to confluence, and serum starved for a minimum of 3 hrs. The media was then changed to either fresh serum free media, serum free media with 100 ng/ml recombinant active two-chain HGF (R&D Systems), or pro-HGF with or without IN-1. To determine the effect on activation of c-Met and the downstream signaling molecules, Gab1 and AKT, the cells were lysed in ice cold RIPA lysis buffer (150 mM NaCl, 50 mM Tris, 1% NP-40, 0.1% SDS, pH 7.4) with protease inhibitor cocktail and Sodium Orthovanadate (Sigma). Activation levels of c-Met, Gab1, and AKT were determined by assessing the levels of the phosphorylated forms relative to the level of the total amount of the specific signaling molecules by western blot analysis.

### Western blot analysis

The protein concentration in cell lysates was determined by BCA assay (Pierce, Rockford, IL) and lysates were separated by 4–12% reducing SDS-PAGE and blotted onto polyvinylidene difluoride (PVDF) membranes (Invitrogen). The following primary antibodies were used for detection: rabbit anti- matriptase (CalBioChem, Philadelphia, PA), rabbit anti-phospho cMet (Y1234/1235), anti-phospho Gab1 (y627), anti-Gab1, anti-phospho AKT (S473), anti-AKT (C73H10), mouse anti-human c-Met (25H2) (all from Cell Signaling Technology), and mouse anti-beta-actin (Sigma). For detection, secondary antibodies conjugated with either alkaline phosphatase (Sigma) or horseradish peroxidase (Chemicon, Temecula, CA) in combination with either the chromogenic substrate nitro-blue tetrazolium and 5-bromo-4-chloro-3′-indolyphosphate (Roche, Indianapolis, IN) or Super-SignalWest Femto Chemiluminescent Substrate (Pierce, Rockford, IL) were used.

### 3D cell culture

The 3D protocol described in [18] was used. Briefly, 1 × 10^4^ cells were plated on 12 mm circular cover glasses coated with Matrigel Matrix Growth Factor Reduced (MMGFR) (BD Biosciences) with serum free Mammary Epithelial Cell Growth Medium (MEGM™) media (Lonza, Walkersville, MD) containing the BulletKit™ growth supplement (BPE, hydrocortisone, GA-1000, Insulin). An overlay of MEGM growth media containing 2% MMGFR was added 1 hr after seeding. Spheroids were allowed to form overnight before addition of 100 ng recombinant HGF (R&D Biosystems) or pro-HGF conditioned media for 24 hrs after which cells were stained with 5 uM Cell Tracker Orange (Invitrogen) for 45 min and Hoechst 33342 (Life Technologies Carlsbad, CA) for 5 min prior to imaging. Confocal images were acquired on the Zeiss LSM 780 scope at the Microscopy Imaging and Cytometry Resources Core at Wayne State University School of Medicine.

### Proliferation assays in human IBC cell lines

30,000 IBC cells were seeded in 6-well plates and serum starved overnight before stimulation with 100 ng active HGF or pro-HGF for 24 h. Cells were then trypsinized and recounted using a hemocytometer to determine the cell number upon stimulation. In the matriptase inhibition assay using IN-1, the synthetic inhibitor was added concomitantly with active HGF or pro-HGF.

## SUPPLEMENTARY MATERIALS FIGURES



## Abbreviations

IBC: inflammatory breast cancer
IHC: immunohistochemistry
IRB: institutional review board
PR: progesterone receptor
ER: estrogen receptor
HER2: human epidermal growth factor receptor 2
H&E: hematoxylin and eosin
HGF: hepatocyte growth factor
TNBC: triple negative breast cancer
