# Supplementary material for: Matriptase regulates c-Met mediated proliferation and invasion in inflammatory breast cancer
Source: Oncotarget. 2016 Aug 12;7(36):58162–73. doi: 10.18632/oncotarget.11262 (PMC5295421; doi:10.18632/oncotarget.11262)
Supplement: Supplementary file 1 [file oncotarget-07-58162-s001.pdf]

Matriptase regulates c-Met mediated proliferation and invasion in inflammatory breast cancer

Supplementary Materials

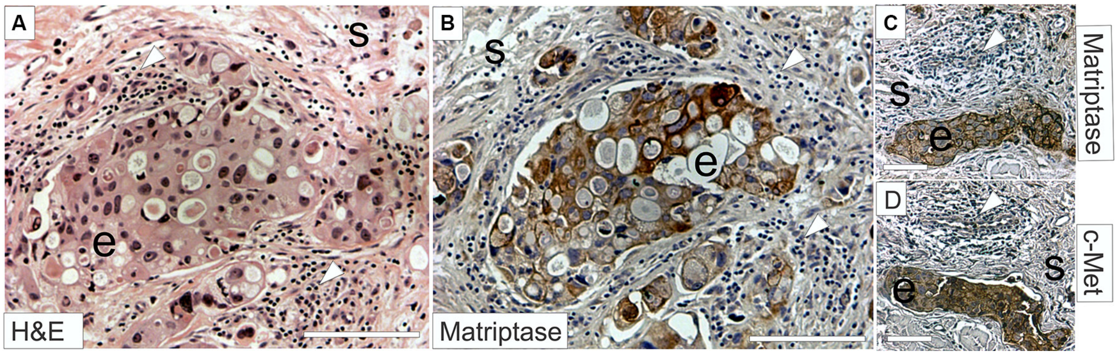

Supplementary Figure S1: Matriptase and c-Met protein expression in areas with inflammation. Representative serial sections of human IBC. (**A**) H&E staining of IBC with inflammation (primarily lymphocytes). IHC using a rabbit anti-matriptase antibody (**B**, **C**) or a rabbit anti c-Met antibody (**D**) Matriptase, and c-Met are primarily localized on cell surfaces (brown staining) of invasive epithelia derived IBC cells (indicated with 'e') with no significant staining in the mesenchymal/stromal compartments (indicated with 's') and display highly similar expression patterns. Inflammatory areas are indicated with arrowheads. IHC tissues were counterstained with haematoxylin (blue/grey). Scale bars, 50 µm.

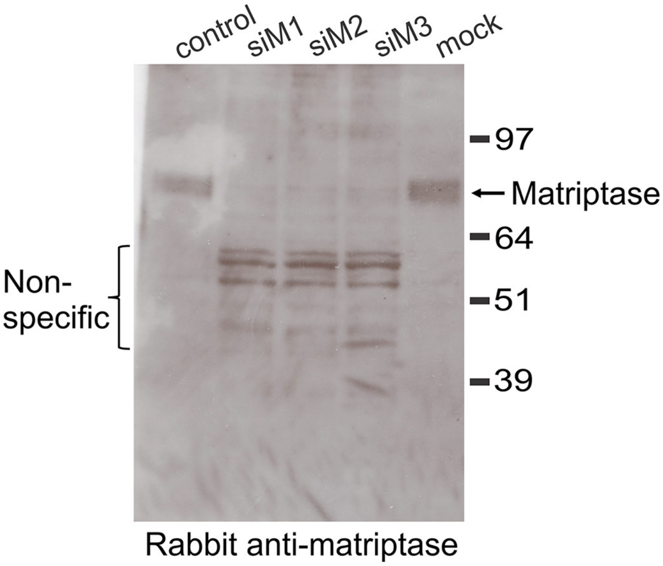

Supplementary Figure S2: Matriptase knock-down in SUM149 cells. Full PVDF membrane showing RNAi silencing of matriptase in SUM149 cells with three independent non-overlapping synthetic RNA duplexes (siM1, siM2, and siM3). A %GC matched RNA duplex was used as negative control as well as a mock where no RNA duplex was added. Rabbit anti-matriptase (Calbiochem/EMD Millipore, San Diego, CA) was used for detection.
